# Sudden-Onset Back Pain and Intermittent Dyspnea While Eating: A Case of Pediatric Achalasia

**DOI:** 10.7759/cureus.56663

**Published:** 2024-03-21

**Authors:** Avrohom Levy, Jennifer Jimenez, Shefali Shah, Mark Kayton

**Affiliations:** 1 Pediatrics, Jersey Shore University Medical Center, Neptune, USA; 2 Pediatric Gastroenterology, Jersey Shore University Medical Center, Neptune, USA; 3 Gastroenterology, Jersey Shore University Medical Center, Neptune, USA; 4 Pediatric Surgery, Jersey Shore University Medical Center, Neptune, USA

**Keywords:** lower esophageal sphincter, esophageal surgery, esophageal motility disorder, esophageal manometry, heller’s myotomy with dor fundoplication, severe achalasia, heller myotomy, pediatric gastroenterology, achalasia, pediatric onset achalasia

## Abstract

A 14-year-old girl with a history of asthma was hospitalized because of sudden-onset back pain around her thoracic region that spread to her chest and abdomen. She had been experiencing dysphagia and breathing difficulties for two years, especially after overeating, which often resulted in vomiting undigested food. CT imaging revealed a severely dilated esophagus narrowing at the gastroesophageal junction, suggestive of type 1 achalasia. Further testing confirmed the diagnosis, with an esophageal manometry showing a lack of esophageal contractions and sphincter relaxation. She then underwent a laparoscopic Heller myotomy with relief to her symptoms. This case underscores the rarity of pediatric-onset achalasia with significant esophageal dilation and secondary airway compression, presenting with unusual musculoskeletal and respiratory symptoms. Timely diagnosis and treatment are crucial to prevent worsening and complications.

## Introduction

Achalasia is a neurodegenerative esophageal motility disorder ordinarily characterized by progressive symptoms of dysphagia, regurgitation, and anorexia [1]. This occurs because of impaired lower esophageal sphincter relaxation and absent or spastic contractions in the esophageal body. This is thought to occur from the degeneration of the myenteric plexus and vagus nerve fibers of the lower esophageal sphincter [2]. The impaired innervation of the esophagus then causes slow or absent bolus transit, which leads to the classic clinical symptoms. In infants, achalasia can present as faltering growth (previously called failure to thrive) because of this process [1].

Achalasia is rare in children, with the incidence of pediatric-onset achalasia ranging from 0.10 to 0.18 per 100,000 individuals [1]. The disease is even less common in children less than five years of age, with most pediatric-onset achalasia occurring in children over 15 years of age. The disease is more prevalent in males and is most commonly idiopathic [3].

The classical symptoms of achalasia typically involve dysphagia, regurgitation, and, occasionally, chest pain and weight loss [2]. The following case will discuss a child who did not complain of these symptoms and, instead, presented with back pain and breathing difficulties.

## Case presentation

**Figure 1:**
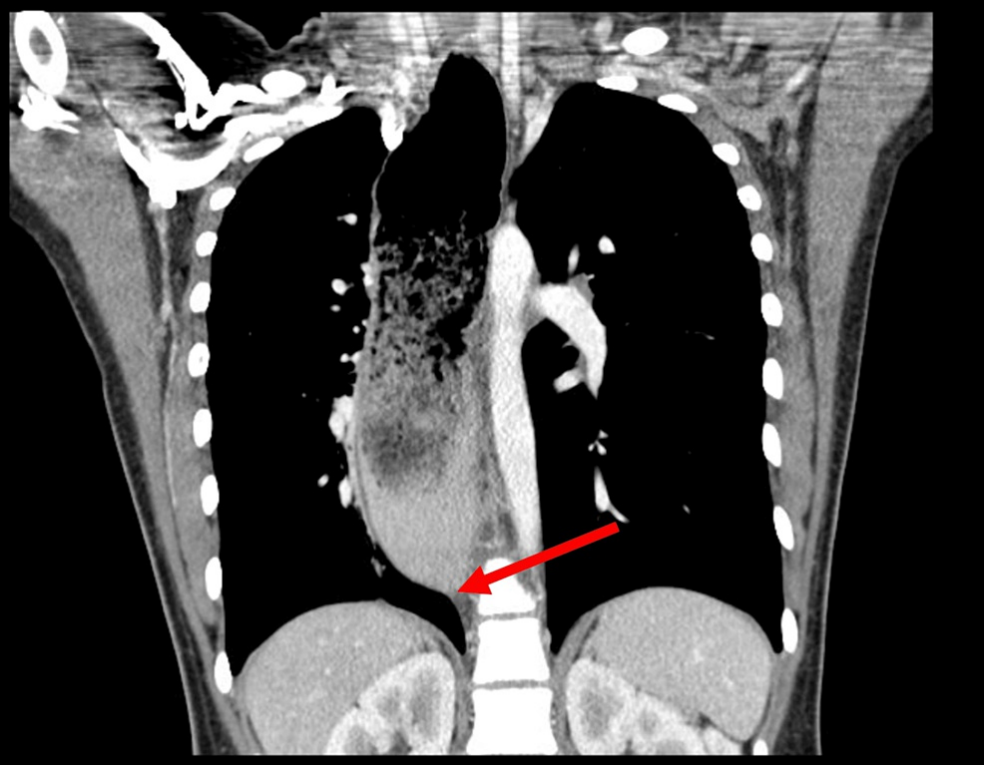
CT Chest Abdomen and Pelvis The red arrow points to the tapering of the esophagus in the gastroesophageal junction region

A 14-year-old female with a past medical history of asthma was admitted to the hospital for sudden-onset back pain around her thoracic region with radiation to her chest and abdomen. The patient was normotensive and afebrile on admission. The patient had a normal body habitus. The pain was initially only felt in her mid to upper back and then began to be associated with lower abdominal pain as well. The patient was nauseated as well. While the patient was waiting in the emergency department, the pain resolved. An ultrasound of the abdomen was suspicious for appendicitis, and the patient was admitted to the Pediatric Surgery Service. A radiograph of the chest showed visible esophageal dilation. Because of the patient’s symptoms and findings found on imaging, a CT chest abdomen and CT pelvis were ordered. The CT imaging showed no evidence of appendicitis but did highlight a markedly dilated esophagus that tapered in the gastroesophageal junction region (Figure 1).

Because of these findings, a further history revealed that over the past two years, the patient did have intermittent issues with difficulty swallowing. The patient would occasionally have trouble swallowing meat and would have to drink a lot of water to facilitate meat passing through her esophagus. The patient would also have trouble breathing if she ate too much. The patient also reported that she would often vomit undigested food.

During hospitalization, the patient was able to tolerate both liquids and soft solids. She was noted to be taking sips of water in between thicker liquids or food. The patient mentioned how she was able to differentiate when foods would "go down into her stomach" or “slowly move down” versus “just sit there [in her esophagus].”

The patient was discharged home and was asked to follow up with an outpatient for an esophagram, an upper endoscopy, and esophageal manometry. In the meanwhile, the patient was recommended only to drink liquids or Ensure until follow-up.

**Figure 2:**
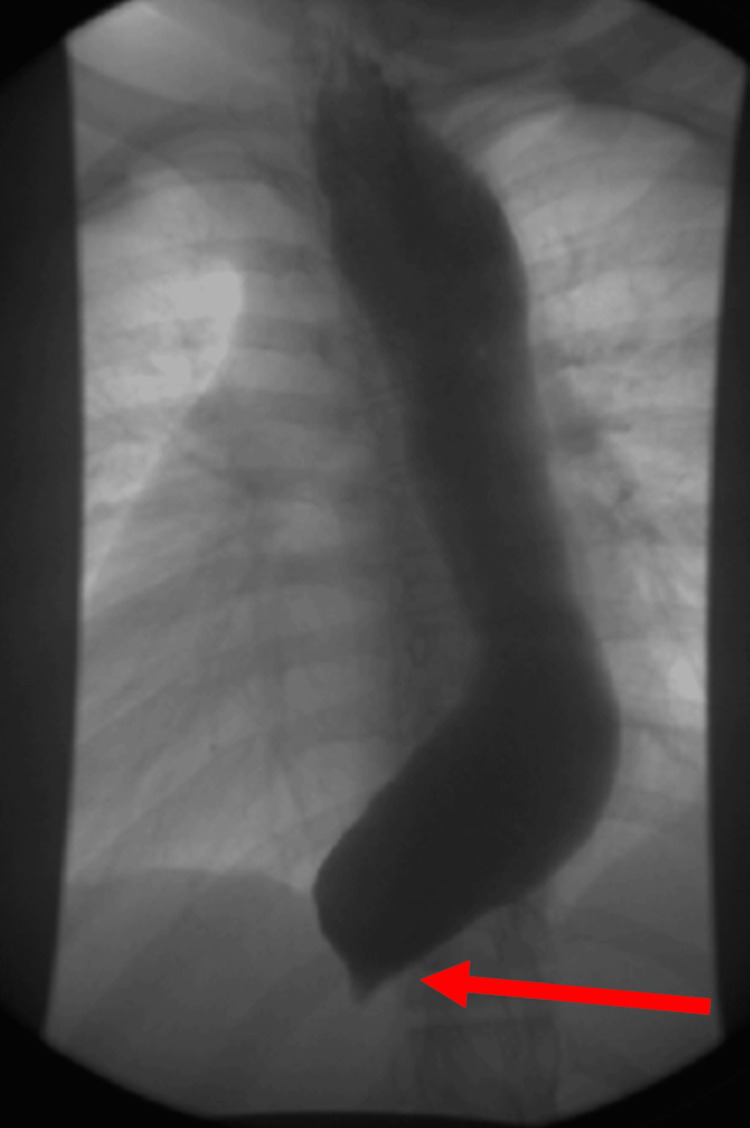
X-ray Esophagogram with Contrast The red arrow points to the contrast failing to progress past the gastroesophageal junction, creating tapering near the gastroesophageal junction

The esophagram was performed and demonstrated findings consistent with achalasia. The study showed diffuse dilation of the esophagus as well as diminished contractility and severe stasis of contrast throughout. There was abrupt tapering found near the gastroesophageal junction with associated failure of contrast to progress past the gastroesophageal junction during the fluoroscopic portion of the examination (Figure 2).

The upper endoscopy was performed under general endotracheal anesthesia, as the patient was at risk for aspiration of possible food contents that may have been retained in the esophagus. During the procedure, the following was noted:

1. The proximal esophagus had normal caliber and normal mucosa. 2. At approximately 20 cm from the lip, the esophagus appeared dilated with liquids observed (no retained foodstuff) that were suctioned. 3. From 20 cm to 40 cm from the lip, the esophagus was very dilated, and the mucosa appeared granular. No trachealization or furrowing was observed. 4. The lower esophageal sphincter appeared closed and pinpointed. Air was insufflated towards the sphincter, which allowed for further advancement of the scope into the stomach.

Biopsies were taken during the endoscopy and showed normal esophageal mucosa in the proximal and distal esophagus. The esophageal manometry showed findings consistent with type 1 achalasia (i.e., a lack of motility in terms of contraction and relaxation of the lower esophageal sphincter), which causes failed peristalsis without abnormal pressurization (Tables 1-2).

**Table 1:** Esophageal Manometry: Lower and Upper Esophageal Sphincter Pressures

Lower Esophageal Sphincter Pressures	
Basal	102 mmHg (normal range 13-43)
Residual (median)/integrated relaxation pressure	86.8 mmHg (normal range < 15.0)
		Upper Esophageal Sphincter Pressures	
Basal (mean)	15.6 mmHg (normal range 34-104)
Residual (mean)	-8.7 mmHg (normal range <12.0)

**Table 2:** Esophageal Manometry: Esophageal Motility

Esophageal Motility	
Number of swallows evaluated	15
% failed	100
% ineffective	0
% pan-esophageal pressurization	0
% premature contraction	0
% fragmented	0

With the diagnosis of achalasia, the patient was then scheduled for a Heller myotomy with Dor fundoplication for definitive treatment. After the procedure, the patient began showing clinical improvement just the first day post-operatively. Subsequent follow-up visits revealed sustained improvement, with the patient reporting the resolution of back pain, shortness of breath after eating, and any eating difficulties.

## Discussion

This case presents a pediatric patient initially presenting with back pain, a nonclassical symptom of achalasia, but subsequent CT imaging raised concerns. Further investigations, including an esophagogram showing contrast failure to progress beyond the gastroesophageal junction and upper endoscopy revealing esophageal dilation and constricted lower esophageal sphincter, suggested achalasia. The diagnosis was confirmed via esophageal manometry, demonstrating absent lower esophageal sphincter contraction and relaxation, along with failed peristalsis.

Achalasia has been known by a variety of terms as its original description includes the terms cardiospasm, megaesophagus, diffuse dilatation of the esophagus without stenosis, and idiopathic dilatation of the esophagus. The term “achalasia” was coined by Sir Arthur Hurst in 1915 and was derived from the Greek “α καλαω,” which means a "lack of relaxation" or "failure to relax." However, the symptoms of achalasia were described more than two hundred years before the term was coined. In 1674, the physician Sir Thomas Willis of England described the first documented case of achalasia [4]. The term achalasia being derived from the Greek words for “failure to relax” is very fitting as it has been shown that peristalsis occurs because of inhibitory vagal nerves creating a latency of contraction, which then becomes impaired in achalasia, creating a failure of the esophagus to relax [5].

There are two categories of achalasia: primary and secondary. Primary achalasia is typically idiopathic [6]. The patient described in this case likely had primary achalasia, as no underlying cause was identified. Secondary achalasia is because of diseases that cause esophageal motor abnormalities, which include autoimmune disorders, infectious causes, and genetic predispositions [6]. One of the rare infectious causes of achalasia is Chagas disease, a disease that occurs predominantly in Central and South America. Chagas disease is linked to the protozoan parasite *Trypanosoma cruzi*, which in turn infects humans via triatomine vectors. However, the incidence of Chagas disease has been decreasing over the past decade, and of the people who have Chagas disease, only about 15-20% will develop gastrointestinal symptoms [7,8]. Chagas disease was not considered for the patient in this case because of the absence of a history of traveling to endemic regions.

Interestingly, eosinophilic esophagitis, a condition with a prevalence of approximately 57 per 100,000 persons, has been found to be associated with achalasia [3,9-11]. Eosinophilic esophagitis is an allergic/immune-mediated disease that involves the eosinophilic infiltration of the esophageal mucosa. There is evidence that the eosinophilic infiltration can cause a release of neurotoxic products, which likely plays a role in achalasia pathogenesis [10]. In this case, biopsies from the upper endoscopy revealed a normal mucosa of the proximal and distal esophagus, effectively ruling out eosinophilic esophagitis as a potential cause.

Diagnosis of achalasia is made difficult by the fact that achalasia generally has an insidious onset. Many patients are first diagnosed with gastroesophageal reflux disease and treated as such, which often goes on for years, prior to a conclusive diagnosis of achalasia [12].

The standard workup for patients presenting with symptoms consistent with achalasia typically includes endoscopy, esophagogram, and esophageal manometry. Endoscopic findings suggestive of achalasia may include a dilated esophagus, food retention, and a tight lower esophageal sphincter during the passage of the endoscope. Esophagogram findings indicative of achalasia may show severe dilation of the esophageal body, often resembling a "bird’s beak" appearance or delayed esophageal emptying in milder cases. Esophageal manometry, considered the gold standard for diagnosing achalasia, utilizes the Chicago Classification to classify achalasia into one of three subtypes based on the absence of peristalsis and impaired relaxation of the lower esophageal sphincter in response to swallowing. Type 1 achalasia is characterized by an abnormal integrated relaxation pressure (IRP) and 100% failed peristalsis, the hallmark features of achalasia. Types 2 and 3 achalasia also exhibit abnormal IRP and 100% failed peristalsis but with additional features. In type 2 achalasia, over 20% of swallows show pan-esophageal pressurization, while in type 3 achalasia, more than 20% of swallows exhibit premature or spastic contractions [12]. In this case, the patient showed no abnormal pan-esophageal pressurization or premature contractions (Table 2), but did display an abnormal IRP (Table 1) and 100% failed peristalsis (Table 2), thus confirming the diagnosis of type 1 achalasia.

While medicine has advanced considerably since the 1600s, current treatments still cannot reverse the underlying neuropathology. Instead, treatments today are aimed at symptom alleviation. Management typically involves medication, chemical paralysis of the lower esophageal sphincter, mechanical or pneumatic dilation, or endoscopic or surgical esophagomyotomy, also known as Heller myotomy, with or without fundoplication [13]. In this case, the patient had a Heller myotomy with fundoplication and displayed marked improvement in her symptoms.

This case is significant as back pain and difficulty breathing are not common presenting symptoms of achalasia, especially in a child. The onset of symptoms in patients is typically insidious, and many patients experience symptoms for years before requesting medical attention [6]. It is likely based on the marked esophageal dilation seen in imaging that the dilation compressed the nearby structures and neurovasculature, causing the presenting symptoms.

Although rare, achalasia leading to airway compression is documented. There have been case reports documenting respiratory complications of mega-esophagus found in the older adult population. Patients with mega-esophagus and airway compression typically have symptoms that worsen after eating. What complicates the issue for these patients is that they are sometimes misdiagnosed initially as having asthma [14]. Fortunately, in this case, the patient received an accurate diagnosis and benefited from appropriate treatment, easing her symptoms.

## Conclusions

Pediatric-onset achalasia with secondary airway compression is an extremely rare occurrence. When a pediatric patient presents with unusual but concerning symptoms such as sudden-onset back pain and intermittent dyspnea with eating, it is important to consider a broad differential that includes achalasia so that this is not missed. In this case, a detailed history did reveal that the patient did in fact have classical symptoms of achalasia. Prompt treatment can prevent worsening symptoms and complications, although in this case, the patient only presented after there was airway compression.
